# Validation and Feasibility of the Medication Acceptability Questionnaire to Investigate Tablet and Liquid Alendronic Acid with Older Hospital Patients

**DOI:** 10.3390/pharmacy6030084

**Published:** 2018-08-11

**Authors:** Sion Scott, Allan Clark, Helen May, Debi Bhattacharya

**Affiliations:** 1 School of Pharmacy, University of East Anglia, Norwich, Norfolk NR4 7TJ, UK; sion.scott@uea.ac.uk; 2 Norwich Medical School, University of East Anglia, Norwich NR4 7TJ, UK; allan.clark@uea.ac.uk; 3 Norfolk and Norwich University Hospital NHS Foundation Trust, Norwich, Norfolk NR4 7UY, UK; helen.may@nnuh.nhs.uk

**Keywords:** medication adherence, bisphosphonate, preference, side effect, alendronate

## Abstract

The effects of formulation characteristics on acceptability are poorly understood. This study evaluated the validity and feasibility of using the Medication Acceptability Questionnaire (MAQ) to investigate factors influencing acceptability of tablet compared with liquid alendronic acid. Written consent was obtained from eligible patients on Older People’s Medicine wards. MAQ face and content validity were evaluated through cognitive interviews while internal consistency and criterion validity were investigated by calculating Cronbach’s alpha and correlation of MAQ items with visual analogue scale (VAS) responses. MAQ data were obtained from 33 and 25 participants for tablet and liquid formulations respectively. Cognitive interviews indicated MAQ face and content validity. The domains of appearance, efficacy, and tolerability demonstrated adequate internal consistency and suitable refinements were identified for the domains of convenience and taste with Cronbach’s alpha <0.7. Significant positive correlations were identified between all MAQ domains and VAS. The liquid trended towards performing better for taste, appearance and tolerability and the tablet for convenience and efficacy. It is feasible to capture patient acceptability of a medication by questionnaire. Interpatient variation in acceptability for two formulations indicates that medication characteristics should be considered during prescribing and medication reviews to match patient preference with the appropriate formulation.

## 1. Introduction

Over 40% of UK women aged over 70 years have osteoporosis [1] for which oral, once weekly bisphosphonate therapy with alendronic acid 70 mg tablet is standard UK prescribing practice. Unfortunately, 50% of patients at a high risk of a fracture discontinue their bisphosphonate within four weeks of starting [2,3,4]. This non-adherence increases fracture risk and avoidable healthcare costs [4,5].

Non-adherence is often an amalgam of intentional and unintentional factors. Unintentional non-adherence arises from factors beyond the patient’s control such as confusion, forgetfulness and physical impairments [6]. There is a consistent causal relationship between dysphagia and unintentional non-adherence [7,8]. The incidence of dysphagia increases with age, ranging from 15% for independently living older people to 68% for the institutionalized [9,10]. Consequently, a significant proportion of people with osteoporosis who are prescribed tablets may find them difficult to swallow.

The liquid formulation of the bisphosphonate alendronic acid, may provide a suitable alternative to such patients and therefore reduce the likelihood of unintentional non-adherence.

Intentional non-adherence arises when the prescribed medication is not acceptable to the patient. Once daily dosing is generally more acceptable and associated with increased adherence relative to multiple dosing regimens [11,12]. However, there are reports of alendronic acid treatment cessation initiated by the patient due to experiences of gastro intestinal side effects and the word ‘acid’ in the medication’s name causing concern [3]. There is substantial research investigating how these factors influence adherence but little information regarding other medication related factors such as formulation characteristics.

The availability of a liquid formulation of alendronic acid may address some of the unintentional and intentional factors associated with bisphosphonate therapy non-adherence. An observational retrospective study (*n* = 375) comparing different bisphosphonates including liquid alendronic acid reported significantly greater persistence with the liquid relative to weekly, monthly, and bimonthly formulations of bisphosphonates. As the study compared different bisphosphonates, the effect of formulation alone could not be isolated [13].

The Medication Adherence Report Scale (MARS^®^) is a validated tool for measuring self-reported adherence. MARS^®^ comprises the five medication non-adherence behaviors of forgetting, altering dose, stopping, missing a dose, and taking less. Each of these behaviors is rated on a five-point Likert scale ranging from always to never [14]. No such validated tool is available for measuring acceptability to medication.

The Medication Acceptability Questionnaire (MAQ) is a newly developed tool to measure patient acceptability to medication formulations [15]. The MAQ is derived from a systematic review of formulation characteristics influencing medication acceptability [16]. This methodological approach to MAQ development affords some confidence in content validity [17]; however further investigation of its psychometric properties is required. MAQ comprises 20 items grouped under the five key domains of medication acceptability identified from the systematic review [16]: convenience (5 items); taste (4 items); appearance (6 items); efficacy (3 items); tolerability (2 items). Each of the items is rated by the respondent on a five-point Likert scale ranging from strongly agree to strongly disagree. Global acceptability with respect to each of the five domains is captured on a 10-point visual analogue scale (VAS). An indication of global formulation acceptability is provided by a final 10-point VAS. The MAQ is available at the following website: http://www.uea.ac.uk/pharmacy/research/maq and is provided in Supplementary File S1.

The present study aims to investigate the psychometric properties of the MAQ and feasibility of using the tool within a trial environment. The study also aims to explore any trends between alendronic acid formulation and, patient acceptability and adherence. The sample size required should a future definitive trial be appropriate will also be estimated.

## 2. Materials and Methods 

### 2.1. Ethics Approval

Ethical and research governance approval were obtained from the London Bromley ethics committee and Norfolk and Norwich University Hospital respectively.

### 2.2. Questionnaire Testing and Refinement

Face and content validity of the Medication Acceptability Questionnaire (MAQ) were assessed through cognitive interviews with patients of Older People’s Medicine wards prescribed tablet alendronic acid. Cognitive interviewing is a form of verbal reporting that allows evaluation of how the target audience perceives survey instruments and their constructs. How a participant interprets a question, processes the information, applies information stored in memory and prepares a response are captured as verbal data during the cognitive interview process. Data gathered may be used to identify survey flaws and improve questions prior to administration in a study [18,19].

Participants in the presence of a research nurse, were asked to concurrently verbalize their thought processes as they completed the MAQ and the research nurse documented any difficulties encountered with understanding, processing, or responding to questions. After completing the MAQ, the research nurse further explored identified difficulties through verbal probing. Where appropriate, participants were asked to suggest improvements to elements of the MAQ that presented difficulties.

Cognitive interviews were continued until no further adaptations were necessary. Participants were also asked whether any factors influencing medication acceptability were unrepresented in the MAQ. The MAQ was iteratively refined to enhance interpretation and ease of response, indicating face and content validity [19].

### 2.3. Study Sample and Setting

#### 2.3.1. Eligibility

Inpatients from an Older People’s Medicine ward regularly prescribed once weekly alendronic acid tablet were eligible for recruitment into the study with the following exclusions:

- Deemed by the healthcare team as unable to provide written, informed consent
- Unable to self-administer medication during in-patient stay or unlikely to self-administer post discharge
- Dysphagia (Functional Oral Intake Scale score <7) [20]

#### 2.3.2. Sample Size

As a feasibility study, neither sample size estimation nor statistical comparisons between the tablet and liquid formulation were undertaken. Sample sizes of between 24 and 50 participants have been recommend for feasibility studies [21,22]. Accordingly, the present study aimed to recruit to within this range.

Dispensing data at the hospital research site suggest a minimum of 20 Older People’s Medicine ward patients per month will be prescribed tablet alendronic acid. Based on a conservative estimate of 30% patient eligibility for study participation and consent, over a 6-month recruitment period, data for 36 participants will be available for analysis.

### 2.4. Recruitment and MAQ Administration

Patients were screened for eligibility and approached for inclusion by a research nurse. Written, informed consent was obtained prior to study enrolment. Participant demographic characteristics plus the following were recorded:

- History of swallowing difficulties
- Manual dexterity assessed by observing participant’s ability to a bottle of liquid alendronic acid and the Hand Grip Strength Test [23]
- Cognitive function assessed by the Abbreviated Mental Test Score [24]

Tablet acceptability and self-reported adherence were captured by participants completing the MAQ and the MARS^®^ [14] respectively based on their existing experiences of taking tablet alendronic acid. A four-week supply of the licensed once weekly liquid alendronic acid was then provided instead of the tablet. This was dispensed as four 100 mL bottles each providing the once weekly dosage of 70 mg alendronic acid. A target of 14 days post liquid initiation was set for re-contacting participants to collect liquid MAQ and MARS^®^ data. The actual date of re-contact was recorded to capture any variation in duration of liquid use. Additionally, for the liquid formulation, medication possession ratio was calculated by inviting patients to report the remaining quantity of liquid alendronic acid bottles. 

### 2.5. Statistical Analysis and Psychometric Testing

Descriptive statistics were calculated for swallowing, manual dexterity, and cognitive function. Each item of the MAQ was scored from 1 indicating ‘strongly disagree’ through to 5 indicating ‘strongly agree’. For each of the five MAQ domains, the item scores were summed giving a domain score. For the domain of tolerability, the items were negatively phrased thus data were inverted for comparison. Domain scores were reported as percentages of the total possible score to adjust for domains having different numbers of items. The domain VAS score ranging from 0 indicating very low acceptability to 10 for maximum acceptability was reported for each of the five domains. Mean and 95% confidence intervals [95% CI] or median and interquartiles (IQ) were reported as appropriate to describe VAS global acceptability of the tablet and liquid formulations. MARS^®^ data were handled similarly; MARS^®^ scores range from 5 to 25 and any deviation from 25 was classified as non-adherence [14].

Regression analysis was performed to identify which of the MAQ domains predict global formulation acceptability. This was based on a two-stage approach. Firstly, the correlation between each domain and acceptability was calculated and then a multiple linear regression model including all significant domains was estimated. This enabled identification of the subdomains independently associated with acceptability. If the residuals were not normally distributed, then the non-parametric bootstrap was used.

Tablet and liquid formulation data were combined to investigate MAQ psychometric properties. Internal consistency of items within each of the five domains was assessed using Cronbach’s alpha, with α ≥ 0.7 deemed acceptable [25]. Spearman’s or Pearson’s correlations were performed to investigate domains with poor internal consistency to identify the items performing poorly and thus requiring adaptation or removal.

Criterion validity was investigated by exploring the relationships between domain subscale scores and their corresponding VAS using Spearman’s and Pearson’s correlation coefficients as appropriate. Any weak or inverse relationships identified between the subscale scores and VAS would suggest that one or more of the items are not capturing the intended attitudes towards the medication.

An estimate of the sample size necessary for a study powered to identify difference in acceptability between a tablet and liquid formulation of alendronic acid was also calculated.

## 3. Results

### 3.1. Questionnaire Testing and Refinement

After three cognitive interviews with patients, no recommendations for improving understanding of the MAQ statements were identified as all reported that the MAQ was easy to answer. All three participants reported that it was helpful in making them think about the characteristics of their medication; they used the terms “very informative”, “makes you wonder why” and “a good and helpful thing”.

No additional factors potentially influencing acceptability not already present in the MAQ were proposed. As face and content validity were demonstrated, no changes were made to the MAQ and no further interviews convened. Cognitive interview participants were female and aged 77 to 85 years. None reported any swallowing difficulties, nor did any have cognitive impairment, one participant reported difficulty with opening sealed boxes.

### 3.2. Recruitment

**Figure 1:**
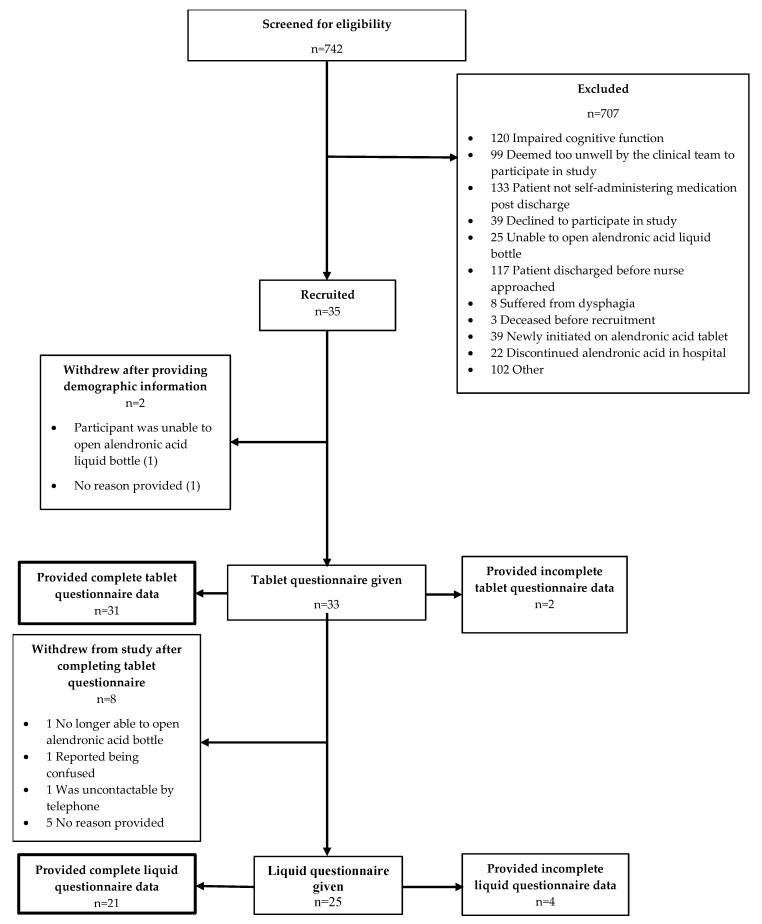
Participant recruitment flow chart.

Figure 1 summarizes participant flow throughout the study. Of the 742 patients screened for inclusion, 35 (5.27% ± 1.67) were recruited. The primary reasons for exclusion were not able to self-administer medication, impaired cognitive function and the patient being discharged before they could be approached. A total of 35 patients consented and provided demographic information; however, 2 (5.7%) participants withdrew before completing the tablet questionnaire. Of the remaining 33 participants who completed the tablet questionnaire, eight (24.2%) withdrew from the study before completing the liquid questionnaire. Data for 25 liquid questionnaires were returned, which was within the target sample size range; however, four (16.0%) were incomplete.

The median (IQ) age for the 33 participants who provided tablet questionnaire data was 84.0 years (78.5, 88.0), of which 28 (84.8%) were female. Participants were prescribed a median (IQ) of 9 (6.0, 11.0) medicines. Abbreviated Mental Test Scores were available for 32 participants and the median (IQ) score was 10 (9, 10), indicating no cognitive impairment [26]. The median (IQ) dominant hand grip test result for males was 14.0 kg (11.0, 18.0) [normative mean = 33 kg] and 10 kg (5.3, 14.0) [normative mean = 20] for females [23]. A history of difficulty with swallowing tablets or accessing them from packaging was reported by 3 (9.1%) of the 31 participants who provided information. The median (IQ) duration between administration of the MAQ for the tablet and liquid formulations of alendronic acid was 14 (12.5, 20.0) days. As the liquid alendronic acid supply was provided immediately after MAQ administration for tablet acceptability, the median (IQ) duration of liquid alendronic acid treatment was also 14 (12.5, 20.0) days.

### 3.3. Questionnaire Responses and Psychometric Testing

Table 1 provides the Cronbach’s alpha values for each of the five MAQ domains. The items of most domains perform well except taste. Further investigation of the relationships between the three item scores for taste and the VAS score for taste, indicates that the item ‘the medicine has no aftertaste’ only has a weak positive Spearman’s correlation coefficient with the other two items in this domain: ‘the medicine tastes good’ and ‘the medicine has a good texture’ (R = 0.267 and 0.31 respectively). Both correlations are significant at *p* < 0.05. The domain of convenience is also slightly below the accepted α threshold of 0.7. The item “is suitable to take when not at home” had a non-significant Spearman’s correlation coefficient with the items “has a convenient dose frequency” and “is a convenient amount for me to take” (R = 0.1 and 0.2 respectively). 

Table 1 also summarizes the relationship between the domain subscale scores and the corresponding domain VAS. A strong positive correlation was present in all cases except for the tolerability domain. Spearman’s correlations for the two items in the tolerability domain indicate that both statements only have weak negative correlations with the VAS score for the domain: ‘the medicine makes me feel ill all of the time’ (R = −0.28, *p* < 0.05) and ‘the medicine makes me feel ill for a short time just after taking it’ (R = −0.40, *p* < 0.01).

**Figure 2:**
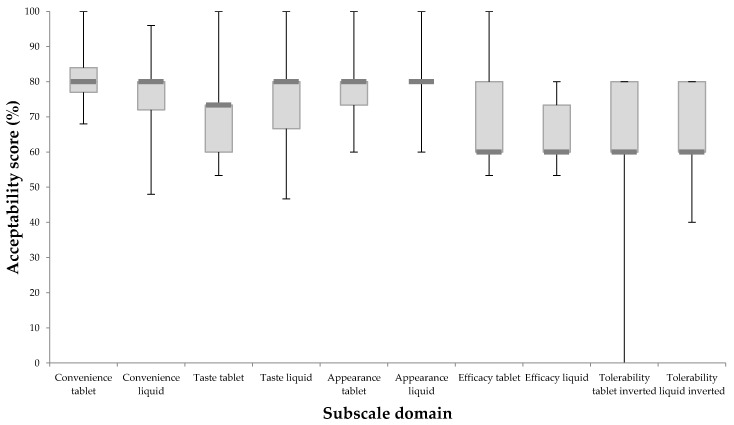
Summary of subscale domain responses for tablet and liquid formulations of alendronic acid. A bar denotes the median response, boxes denote the upper and lower quartiles and whiskers indicate the minimum and maximum responses.

Figure 2 illustrates participant responses to the five subscale scores. The median scores for both the tablet and liquid were relatively similar. The MAQ demonstrated ability to discriminate between the two formulations as based on the interquartiles; there was a general trend for the liquid to perform better in terms of taste, appearance, and tolerability and for the tablet to perform better in terms of convenience and perceived efficacy. The statement within the subscale domain of convenience that elicited the greatest difference between tablet and liquid was ‘It is suitable to take when not at home’. The median (IQ) score for the tablet was 4.0 (4.0, 4.0) indicating that at least 75% of participants agreed with the statement. However, the liquid scored 4.0 (2.0, 4.0) indicating that only 50% of the participants agreed with this statement.

MAQ also discriminated between the different subscales as the efficacy and tolerability subscales had scores at around 60% for both formulations while the remaining three subscale domains all had median values above 70%.

The median (IQ) global acceptability score for the tablet was marginally higher than the liquid with 9 (7.5, 10.0) compared to 8 (5.0, 10.0).

Spearman and Pearson correlations between each of the five subscale domain scores and global acceptability are summarized in Table 2. For the tablet, only the subscale domains of convenience and taste demonstrated a significant positive correlation with global acceptability. For the liquid all the subscale domain scores demonstrated a positive correlation with global acceptability and in all cases, except for tolerability, these were significant relationships.

Informed by the statistically significant correlations reported in Table 2, convenience, taste, appearance, and efficacy were entered into a multiple regression analysis; the models are provided in Table 3. The resulting model predicted 56.4% of the variance (R^2^ = 0.564, *p* = 0.001) of tablet global acceptability. The overall VAS score for taste and appearance made a statistically significant contribution to the prediction. A higher taste score predicted a higher global acceptability score (B = 1.212, *p* < 0.001), after adjustment, a lower appearance score was predictive of higher tablet global acceptability (B = −0.678, *p* < 0.001). The model for predicting global acceptability score for the liquid accounted for approximately 67% of the variance (R^2^ = 0.668, *p* < 0.05). The sub scale domains that were statistically significant to the prediction were convenience (B = 0.495, *p* < 0.05) and taste (B = 0.629, *p* < 0.05). With both domains, the higher score predicted a higher global acceptability VAS score.

MARS^®^ median (IQ) score (*n* = 24) for the liquid was slightly higher at 25.0 (24.0, 25.0) compared with 24.0 (24.0, 25.0) for the tablet [14]. Liquid alendronic acid usage data were available for 21 of the 25 participants and all had used enough bottles indicating 100% persistence. If treatment cessation is assumed for the four participants for whom data were unavailable, the most conservative estimate of persistence with liquid treatment was 84%.

The most appropriate primary outcome measure for a future definitive study is tolerability as there is potential for direct clinical benefit if the liquid is better tolerated. The mean (SD) tolerability subscale score for the tablet was 6.21 (1.92) compared with 6.54 (1.35) for the liquid. A difference in subscale score between the two formulations of 0.5 was considered clinically important. The pooled standard deviation across both the tablet and liquid group was 1.6. Assuming a SD of 1.6, to detect a difference of 0.5 in subscale score between the two formulations with 80% power, a sample size of 161 per group and thus a total sample of 322 participants will be required for a definitive parallel group randomized controlled trial.

## 4. Discussion

The high rate of MAQ completion indicates that it is an acceptable tool for use in a trial environment with an older population. The observed variations in acceptability of the tablet and liquid formulation indicate that the MAQ can discriminate between formulations; further work is required to determine whether these results are stable over time through undertaking test-retest reliability analysis. From a clinical perspective, this suggests that it is important for prescribers to involve patients in the decision-making process when initiating or switching between formulations. Acknowledging the limitations of a small sample sized feasibility study, which is therefore not powered to detect statistically significant differences, a trend towards better tolerability and adherence was observed for the liquid formulation while the tablet formulation was perceived to be more convenient by the ambulatory participants of this study. Furthermore, persistence to the liquid at three weeks post discharge was substantially higher than the 50% previously cited for trial participants prescribed the tablet formulation [3]. However, this result should be interpreted with caution as persistence with the liquid formulation was indirectly evaluated by calculating the medication possession ratio, which may not necessarily equate to successful administration. Nonetheless, given the advantages of this positive outcome, a definitive randomized controlled trial to establish whether this effect is maintained in a larger more generalizable patient sample using direct measures of adherence is warranted.

The very low rate of patient refusal to complete the MAQ and try liquid alendronic acid indicates that a future definitive study is feasible. The age and medicine regimen complexity of participants reflects the expected population prescribed alendronic acid, affording some confidence in the likely generalizability of findings [22]. The good accessibility of the liquid packaging to most screened patients contrasts previous research findings with tablet blister packaging which indicates poor accessibility for older people [23]. Despite the participant population having approximately half the grip strength of the average population aged over 70 years, they were all able to successfully open the alendronic acid liquid bottle even though some had reported difficulties with popping tablets out of blister packaging [23].

While most MAQ items demonstrated adequate to excellent internal consistency, it was weaker for some items in the taste and convenience domains. The poor correlation in the taste domain may be due to item ‘the medicine has no aftertaste’ not specifying whether the aftertaste was good or bad [27]. In contrast the two other items in the taste domain explicitly invite respondents to indicate whether the formulation has a “good” taste and texture. The statement may be improved by clarifying that the medicine has no bad aftertaste [28]. For the convenience domain, the item “is suitable to take when not at home” correlated with fitting into lifestyle and being easy to take. The poor correlation with items relating to dose frequency and amount may be due to the suitability of alendronic acid to take when not at home being independent of the frequency and amount. Alendronic acid is very favorable in terms of frequency and amount as it is a once weekly, single dose medication. However, the demanding requirements of administration 30 min before breakfast with a glass of water is impractical when not at home.

The largely strong positive correlations between the domain subscale and VAS scores indicate good criterion validity. It may also be appropriate for just the domain VAS scores to be used in future studies to evaluate patient acceptability to medicine characteristics thus reducing the questionnaire burden from 57 items to 11 items while preserving validity. However, the poor correlation between the tolerability subscale and VAS needs further investigation before the questionnaire can be reduced to 11 items. While the participants were reporting poor tolerability in response to the domain subscale questions, these experiences did not translate into the overall VAS score for the tolerability domain. This indicates that despite experiencing side effects, respondents perceived this to be acceptable.

The small sample size arising from the high rate of patient ineligibility due to not self-administering medication, impaired cognitive function and being discharged before approach is of concern. Recruitment in the primary care environment may provide a physically and cognitively more able population thus overcoming the issue of recruitment prior to hospital discharge. Additionally, duration of prior alendronic acid tablet use by participants could have been collected. This would have enabled reporting of the duration to which the self-reported adherence relates. The present study only reports duration of use for the liquid as the UK hospital setting does not have access to such historical primary care prescribing data hence this was a limitation of the present study.

Order of exposure to tablet followed by liquid may account for some of the variance in the regression model; however, given that the tablet formulation is the standard treatment for osteoporosis in the UK, it was not feasible for an equal number of participants to be first exposed to the liquid rather than tablet formulation.

All participants were ambulatory thus it is unsurprising that one single tablet was considered more convenient than a 100 mL bottle of liquid given that the main driver for this preference was the convenience of being able to take the formulation when not at home. However, all formulations of alendronic acid are taken in the morning before food resulting in most patients administering while at home. This reduces the likelihood of patients being able to realize the stated benefit of tablet transportability. These findings do indicate that the restrictions of alendronic acid administration may present a barrier to adherence and therefore should be discussed at the point of initiation [3].

The trend towards greater perceived efficacy of the tablet is interesting given the asymptomatic nature of osteoporosis resulting in no tangible short-term effects. This perception may be due to patients associating efficacy with experiencing tablet-associated side effects such as indigestion. Alternatively, there may be a perceived hierarchy of medication formulation with tablets considered more effective than liquids. The trend towards greater self-reported adherence to the liquid may be related to observations such as the trend towards improved tolerability with the liquid as side effects of alendronic acid are often cited as a reason for discontinuation [2,24].

This feasibility study has provided information regarding optimal design of a subsequent study and demonstrated the suitability of MAQ for exploring patient perceived acceptability of a medication. It suggests that formulation characteristics should be discussed when making a prescribing decision. The before and after nature of the study prohibits a causal link being attributed to the differences between tablet and liquid acceptability but suggests that further research is warranted.

## 5. Conclusions

The MAQ is suitable for capturing patient perceived acceptability of a medication and demonstrates reasonable psychometric properties which may be improved by further refinements. It has discriminated between a tablet and liquid formulation providing an early indication of the acceptability of liquid alendronic acid relative to the tablet. Medicine formulation characteristics should be considered when making a prescribing decision.

## Figures and Tables

**Table 1:** Relationships between MAQ domain items and, domain subscale scores with their corresponding visual analogue scale (VAS).

MAQ Domain	Internal Consistency (α) between Domain Item Scores	Criterion Validity Spearman’s Correlation Coefficient (*p*-Value) between Domain Subscale Score and Corresponding VAS
**Convenience**	0.65	0.63 (<0.001)
**Taste**	0.56	0.65 (<0.001)
**Appearance**	0.71	0.58 (<0.001)
**Efficacy**	0.81	0.71 (<0.001)
**Tolerability**	0.91	0.37 (0.005)

**Table 2:** Spearman’s correlations between domain subscale scores and global acceptability visual analogue scale (VAS).

Subscale Domain	Correlation Coefficient (*p*-Value)	
	Tablet Global Acceptability VAS	Liquid Global Acceptability VAS
Convenience	0.40 (0.022)	0.63 (0.001)
Taste	0.41 (0.018)	0.64 (0.001)
*Appearance	−0.17 (0.355)	0.44 (0.031)
Efficacy	0.21 (0.243)	0.62 (0.002)
*Tolerability inverted	−0.06 (0.753)	0.20 (0.361)

* Pearson’s correlation.

**Table 3:** Multiple regression models for the tablet and liquid formulations of alendronic acid identifying which of the MAQ domains predict global formulation acceptability.

	Tablet Global Acceptability VAS		Liquid Global Acceptability VAS	
Subscale Domain	Regression Coefficient (95% CI)	*p*-Value	Regression Coefficient (95% CI)	*p*-Value
Convenience	−0.09 (−0.53, 0.34)	0.675	0.50 (0.05, 0.94)	0.032
Taste	1.21 (0.57, 1.86)	<0.001	0.63 (0.13, 1.13)	0.017
Appearance	−0.68 (−1.05, −0.3)	<0.001	−0.05 (−0.56, 0.46)	0.837
Efficacy	0.30 (−0.04, 0.63)	0.077	0.53 (−0.25, 1.30)	0.167
Tolerability inverted	−0.01 (−0.28, 0.26)	0.938	−0.11 (−0.92, 0.70)	0.769
Constant	10.74 (3.94, 17.54)	0.002	−12.77 (−26.66, 1.12)	0.069

## Supplementary Materials

The Medication Acceptability Questionnaire (MAQ) is available online at https://www.uea.ac.uk/pharmacy/research/maq and is provided in Supplementary File S1.
