# Supplementary material for: Validation and Feasibility of the Medication Acceptability Questionnaire to Investigate Tablet and Liquid Alendronic Acid with Older Hospital Patients
Source: Pharmacy (Basel). 2018 Aug 11;6(3):84. doi: 10.3390/pharmacy6030084 (PMC6163684; doi:10.3390/pharmacy6030084)
Supplement: Supplementary file 1 [file pharmacy-06-00084-s001.pdf]

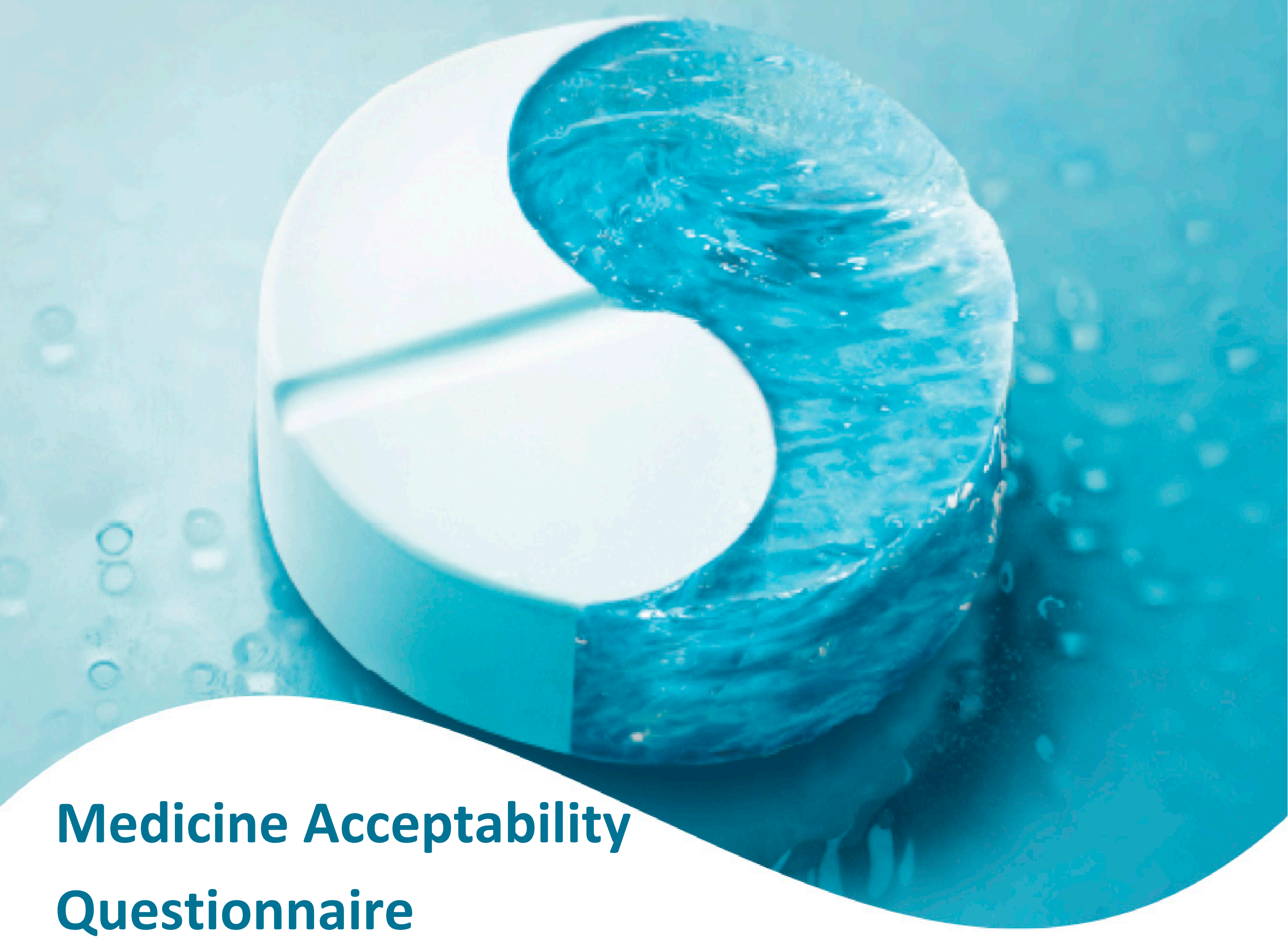

Medicine Acceptability Questionnaire

Some medicines are available in different forms such as tablets, capsules and liquids. We know that patients might prefer one form over another.
This questionnaire will tell us about your preferences for {insert name of medication and formulation under investigation}.

There are six questions for you to answer about medicine characteristics that might affect your preference. The questionnaire should take less than 10 minutes to complete.

The MAQ, is subject to copyright protection owned by University of East Anglia. If you would like to use the MAQ this can be supplied to you under license, for non-commercial use by non-commercial organisations this is supplied free of charge. If you would like to request a MAQ licence please contact Dr Georgina Pope Georgina.pope@uea.ac.uk.

1. How **convenient** is this medicine?

For each of the following statements, please tick the **ONE** box that best describes your opinion.

This medicine…	Strongly disagree	Disagree	Neither agree nor disagree	Agree	Strongly agree
…has a convenient dose frequency. (E.g. once a day, twice a day or once weekly.)	☐	☐	☐	☐	☐
…is a convenient amount for me to take. (E.g. number of tablets or volume of liquid.)	☐	☐	☐	☐	☐
…is easy to fit into my lifestyle.	☐	☐	☐	☐	☐
…is easy to take. (E.g. consider need for taking on an empty stomach or standing upright.)	☐	☐	☐	☐	☐
…is suitable to take when not at home. (E.g. consider need for water, or ease of carrying.)	☐	☐	☐	☐	☐

Please tell us overall how happy you are with the **convenience** of this medicine. Please **CROSS** the number that best describes how you feel ( ).

Very unhappy — Neither — Very happy

0 – 1 – 2 – 3 – 4 – 5 – 6 – 7 – 8 – 9 – 10

2. How does this medicine **taste?**

For each of the following statements, please tick the **ONE** box that best describes your opinion.

This medicine…	Strongly disagree	Disagree	Neither agree nor disagree	Agree	Strongly agree
…tastes good.	☐	☐	☐	☐	☐
…has no aftertaste.	☐	☐	☐	☐	☐
…has a good texture. (E.g. smooth or fizzy.)	☐	☐	☐	☐	☐

Please tell us overall how happy you are with the **taste and texture** of this medicine. Please **CROSS** the number that best describes how you feel ( ).

Very unhappy — Neither — Very happy

0 – 1 – 2 – 3 – 4 – 5 – 6 – 7 – 8 – 9 – 10

3. How does this medicine **look and smell?**

For each of the following statements, please tick the **ONE** box that best describes your opinion.

This medicine…	Strongly disagree	Disagree	Neither agree nor disagree	Agree	Strongly agree
…is easy to swallow (right size, shape, thickness or texture).	☐	☐	☐	☐	☐
…is easy to see.	☐	☐	☐	☐	☐
…has a nice colour.	☐	☐	☐	☐	☐
…has no bad smell.	☐	☐	☐	☐	☐
…is easy to take out of its packaging.	☐	☐	☐	☐	☐
…is easy to hold.	☐	☐	☐	☐	☐

Please tell us overall how happy you are with the **appearance and smell** of this medicine. Please **CROSS** the number that best describes how you feel (☒).

Very unhappy — **Neither** — **Very happy**

0 — 1 — 2 — 3 — 4 — 5 — 6 — 7 — 8 — 9 — 10

4. How well does this medicine **work?**

For each of the following statements, please tick the **ONE** box that best describes your opinion.

This medicine…	Strongly disagree	Disagree	Neither agree nor disagree	Agree	Strongly agree
…does what it is supposed to do. (E.g. helps my symptoms or prevents further illness.)	☐	☐	☐	☐	☐
…works quickly.	☐	☐	☐	☐	☐
…makes me feel better.	☐	☐	☐	☐	☐

Please tell us overall how happy you are how well the medicine **works**. Please **CROSS** the number that best describes how you feel (☒).

Very unhappy — **Neither** — **Very happy**

0 — 1 — 2 — 3 — 4 — 5 — 6 — 7 — 8 — 9 — 10

© University of East Anglia. Contact Georgina.pope@uea.ac.uk if you would like to use the MAQ

5. Does this medicine have **side effects?**

For each of the following statements, please tick the **ONE** box that best describes your opinion.

This medicine…	Strongly disagree	Disagree	Neither agree nor disagree	Agree	Strongly agree
…makes me feel ill all the time.	☐	☐	☐	☐	☐
…makes me feel ill for a short time just after taking it.	☐	☐	☐	☐	☐

Please tell us overall how happy you are with the **side effects** of this medicine. Please **CROSS** the number that best describes how you feel (⊗).

Very unhappy | Neither | Very happy

0 — 1 — 2 — 3 — 4 — 5 — 6 — 7 — 8 — 9 — 10

6. Overall acceptability

Please tell us overall how happy you are with the **acceptability** of this medicine. Please **CROSS** the number that best describes how you feel (⊗).

Very unhappy | Neither | Very happy

0 — 1 — 2 — 3 — 4 — 5 — 6 — 7 — 8 — 9 — 10

Thank you for taking the time to complete this questionnaire.

© University of East Anglia. Contact Georgina.pope@uea.ac.uk if you would like to use the MAQ
